# A Cloud-Based X73 Ubiquitous Mobile Healthcare System: Design and Implementation

**DOI:** 10.1155/2014/145803

**Published:** 2014-03-10

**Authors:** Zhanlin Ji, Ivan Ganchev, Máirtín O'Droma, Xin Zhang, Xueji Zhang

**Affiliations:** ^1^Telecommunications Research Centre (TRC), University of Limerick, Limerick, Ireland; ^2^College of Information, Hebei United University, Tangshan 063009, China; ^3^Research Center for Bioengineering and Sensing Technology, University of Science and Technology, Beijing 100080, China

## Abstract

Based on the user-centric paradigm for next generation networks, this paper describes a ubiquitous mobile healthcare (uHealth) system based on the ISO/IEEE 11073 personal health data (PHD) standards (X73) and cloud computing techniques. A number of design issues associated with the system implementation are outlined. The system includes a middleware on the user side, providing a plug-and-play environment for heterogeneous wireless sensors and mobile terminals utilizing different communication protocols and a distributed “big data” processing subsystem in the cloud. The design and implementation of this system are envisaged as an efficient solution for the next generation of uHealth systems.

## 1. Introduction

As wireless medical sensors are becoming more popular and cloud computing technology is growing up, they provide the condition for the development of a body area sensor network (BASN) component and a cloud environment for the ubiquitous mobile healthcare (uHealth) systems. BASNs are considered to be an important means to relieve the pressure of insufficient medical resources in an aging era and are becoming a strategic direction of the uHealth research. At present, BASN are still at an early developing stage and facing a series of challenges, such as the existence of heterogeneous sensor protocols and “big data” computing and mining. BASN is defined as a wireless network, which is formed by sensors located on, and/or biosensors transplanted into, the human body [1, 2] and a data collector (Sink) used for medical data collection in real time. BASN can gather medical data, perform classified learning, and analyze data in real time, thus realizing an early medical warning [3, 4]. Being a network type of huge significance and demand, BASNs become a strategic direction of the uHealth research.

Researchers show that, at present, there are 1 billion people worldwide with underweight and at least 0.3 billion people with a morbid obesity. By 2015, the number of subhealth world population will reach 1.5 billion, and by 2020, the expense on chronic diseases will reach 10 thousand billion dollars [5]. By 2025, the aging population will reach 1.2 billion and this is a giant challenge to the provision of sufficient medical resources. Aiming at these global problems, changing the way of providing medical services becomes quite important. As long as the unnecessary links in the medical service system are wiped off, current technology can improve the health condition and life quality of the mankind, while at the same time medical resources will be utilized fully and in an optimal way [6]. BASN can solve the problem of shortage of both material and manpower resources in the medical sector effectively by classifying, learning, analyzing, and storing the patients' medical data and by providing the patients with early medical warnings, as a supplement to the traditional medical systems.

The uHealth systems are based on the Internet of Things concept [7], whereby the medical treatment emphasizes the object management. This change of the concept urges the mobile medical technology to adapt. “Things” in the uHealth systems include doctors, patients, medical devices, and sensors. “Join” refers to all things being appreciable, interactive, and controllable. “Network” is the working flow of medical treatment and health management. The uHealth systems are taking shape to a brand-new ecosphere. At this “big data” era, researchers show that the data generated by sensors will reach petabyte (PB) level annually [8], if electronic health recording is set up only for 0.1 billion people. The medical cloud provided by uHealth systems can reduce the cost of medical informatization by providing a foundation for storing and mining of “big data” with high availability, expansibility, and performance of the data storage system; a “big data” management and processing platform which adapts to different needs; a data cleaning and loading mechanism; a real-time data search and complex data analysis package, and so forth. All of these can be efficiently used for disease prevention, health condition monitoring, and timely remedy, as a strategy and a core content of the future uHealth development.

## 2. Related Research

At present, in both industry and academia, a lot of significant work is done in this area. For example, in the CodeBlue medical care project, conducted by the U.S. Harvard Sensor Networks Lab [9], an ad hoc sensor network infrastructure was proposed and developed for emergency medical care, by providing a flexible naming and discovery algorithms to ensure seamless transfer of data in the system. The MiThril NGN wearable research platform, developed by MIT Media Lab [10], is a next generation context-aware system providing human-computer interaction with body worn applications. The MyHeart telemedicine EU FP6 project [11], led by Philips, has developed an intelligent system for the prevention of cardiovascular diseases by periodic monitoring on the vital signs and establishing the health status in real time.

Current research in the BASN area is mainly going in two directions—power saving and communication protocols. In [12], an ultralow-power wireless sensor network solution was proposed. The authors stated that the design of a low-power sensor is very important as the battery lifetime is expected to increase only by 20% in the next ten years. Medical sensors were not mentioned, but from research it is clear that reducing the power consumption is one of the main goals when designing wireless healthcare middleware. In [13], Intel provided an evaluation software kit for the development of embedded devices, which are used to interact with medical devices. It is based on the Windows Mobile operating system, and the devices must be certified by Continua. Libresoft (http://openhealth.libresoft.es/) reported on an open-source ISO/IEEE 11703 Personal Health Data (hereafter it is called X73 [14]) system with its own Bluetooth application programming interface (API). The authors stated that it is not easy to develop a uHealth system operating in a cloud environment. In [15], a uHealth system based on the X73 standards was proposed, but the system seems as not being able to provide a fully ubiquitous solution.

Although the literature reports on a number of valuable results, BASNs are still at an early developing stage. There are many important issues which need to be solved and researched further. As the low-powered processing chips, smart mobile terminals, cloud computing, and NGN develop rapidly, there is a significant opportunity for healthcare system development. Researching on BASN systems upon cloud computing is of important significance to medical informatization. This paper focuses on the development of a cloud-based X73 uHealth system, consisting of a healthcare middleware in the BASN domain and a distributed data processing system in the cloud.

**Figure 1:**
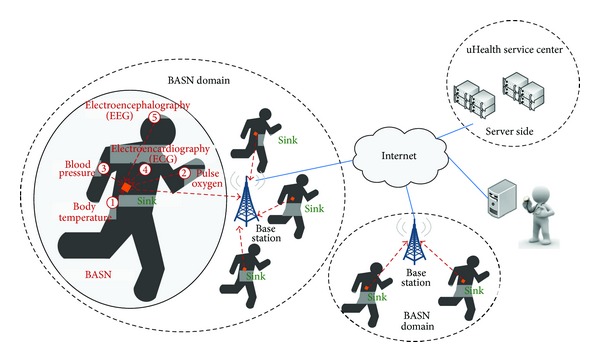
A high-level view of a BASN-based uHealth system.

Figure 1 depicts the high-level view of a common BASN-based uHealth system. Operating as part of the system, sensor nodes obtain and send medical data to a Sink node. After some aggregation in the Sink node, the data is pushed to a medical server for further processing and mining.

The main problem in the BASN domain is related to sensors compatibility. Different vendors may implement different communication protocols into their medical sensors, such as ISO, IEEE, and HL7, each one with its own communication mechanism, data format, and so forth. To solve this problem, the IEEE approved the X73 standard [16], which provides an efficient real-time exchange method for plug-and-play operation. Due to the complexity of the X73 protocols, in the industry, currently only Continua Health Alliance (http://www.continuaalliance.org/) produces a hardware which is fully compatible with this standard. In the academia, Korean scholars have reported that a sensor could be adapted to the X73 protocols by means of an extra hardware [17], but this will increase the cost of the sensor. Developing of software middleware as an adapter is proposed in this paper.

As cloud computing offers a low-cost solution to “big data” storage and processing, a kind of healthcare system enterprise solutions upon cloud computing emerged recently, for example, proposed by IBM, Microsoft, Cisco, and so forth. Most of them, however, are only concerned with the infrastructure, that is, the informatization of hospitals, medical treatment clouds, and so forth. Only few companies are doing research on the BASN-oriented cloud. In the academia, Laleci et al. have proposed an electronic health record system based on cloud computing [18]. Sobhy et al. have put forward the theory of MedCloud [19], but did not propose the application of BASN. Even though these are valuable examples in the area of medical data collection and storage based on cloud computing, BASN, especially the data acquisition technology upon X73 BASN, was not involved in these systems. The problem of processing a massive amount of medical data with higher throughput and lower delay needs to be studied more extensively.

From the view of applications, BASN is with wide prospect. As the aging society is short of medical resources, BASN could offer an effective solution to this problem, which will surely be the core one in the development of future uHealth systems. Further fusion with IoT will lay down the application direction of BASN in the future. For example, BASN-based system can help monitor the eruption of epidemic situation, mass outbreak disease, or common disease in the community, and as such can provide prevention and/or early warning on emergency events. BASN will continue being developed toward smart, micro, and wearable orientation. In the meantime, the establishment of large-scale BASN-based health monitoring platforms utilizing ubiquitous wireless communication techniques is envisaged to take place.

With the X73 standard and cloud computing technology, this paper reports the development of a distributed uHealth system for medical data collection, storage, analysis, and mining with high performance and high reliability.

## 3. X73 BASN-Based uHealth Architecture

**Figure 2:**
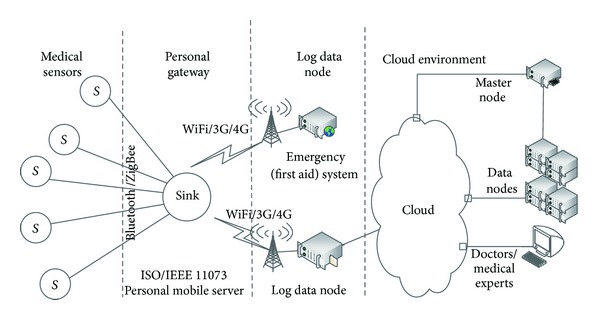
The X73 uHealth system architecture.

The X73 BASN-based uHealth system architecture is shown in Figure 2.

In the Medical Sensors tier, a number of wireless medical sensors (S)—such as pulse oximeter, heart rate monitor, blood pressure monitor, thermometer, weighting scale, and glucose meter—operate in a plug-and-play manner. They use Bluetooth or ZigBee for communication with the ISO/IEEE 11073 based middleware (Sink) in the Personal Gateway tier. The mobile phone of the patient acts usually as such a gateway. A software agent, operating on the phone, periodically collects data from the sensors and sends a personalized message to a log data node and/or emergency system under the Always Best Connected and best Served (ABC&S) communication paradigm [20]. The message contains medical parameters' values, the user's location, medical device's information, IP address, and so forth. In the cloud, a relevant cloud ant collects all such messages by using the defined scheduling algorithms and generates medical-care advices/instructions/statements which are sent back to patients. The medical-care contents are personalized and customized to best suit each mobile phone and patient. In the cloud, a set of data mining applications run for log data collecting, analyzing, processing, and advising. Different patients may receive different medical-care advices and statements based on their personal profiles and current medical condition. Also, the cloud provides medical experts' and doctors' manual diagnosis when needed.

The X73 uHealth system, described in this paper, proposes a brand new environment based on an X73 PHD BASN and a cloud architecture.

### 3.1. BASN

The developed X73 healthcare middleware supports the domain information model (DIM), medical device encoding rules (MDER) and decoding library, and the communication model (CM). DIM defines details of the health device object, medical device system (MDS), and metrics. MDER expresses DIM objects by means of the abstract syntax notation-one (ASN.1) specifications [21]. The service model (SM) defines the object access methods and event reporting. CM states the finite state machine (FSM) for the ISO/IEEE 11073 agent (sensor node) and the ISO/IEEE 11073 manager (middleware). A rule engine is used for separating the business logic from the middleware. A gateway agent acts as a bridge between the personal mobile server and the rest of the uHealth system. The middleware uses a multiagent technology and a sensor adapter software to realize protocol conversion for non-X73 sensors.

**Figure 3:**
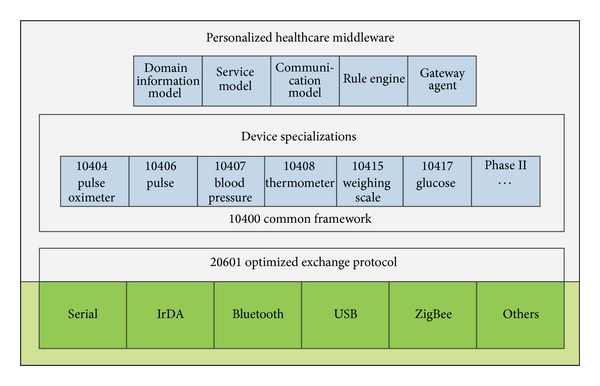
The middleware of the X73 uHealth sensor node.

The middleware architecture of the sensor node is depicted in Figure 3. The communication support layer is at the bottom of this architecture. It is provided by the operating system of the user's mobile phone. The middle layer includes the ISO/IEEE 11073 PHD device specializations. The DIM, SM, CM, rule engine, and gateway agent operate at the top layer.

### 3.2. Cloud Architecture

Based on the cloud computing technology, the designed and developed X73 cloud architecture includes an efficient distributed message system used for medical data collection, a fault-tolerant real-time computing system for data storage, and a Hive system for data mining.

The distributed framework Apache Hadoop is suitable for the design of the X73 uHealth cloud as it provides an efficient Hadoop Distributed File System (HDFS) [22] for data storing and a Hadoop MapReduce [23] for “big data” processing. In order to increase the performance of the cloud platform, this paper introduced a set of components for efficient collection, storage, and analysis of “big data” describing the user's medical condition.

As regards the medical data collection and storage, the traditional data storage way of the O (lg n) B + tree algorithm is not suitable for this system due to its low efficiency. A Publish/Subscribe based distributed message system, supporting two methods of synchronous collecting and asynchronous subscription, is proposed in this paper.

As regards medical data storage and analysis, the high fault-tolerance HDFS provides possibility to store data on a low-cost computer with high aggregate bandwidth across the Hadoop cluster. The Facebook's Hive [24], acting as data warehouse, provides data summarization, query, and analysis in the Hadoop distributed environment.

**Figure 4:**
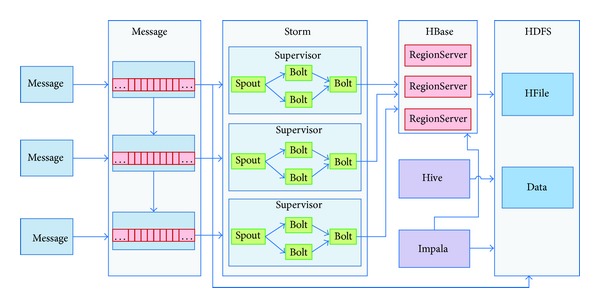
The cloud architecture in the X73 uHealth system.

Based on the Hadoop ecosystem, the X73 uHealth cloud application architecture is shown in Figure 4.

## 4. System Design

### 4.1. Middleware of X73 uHealth Sensor Node

**Figure 5:**
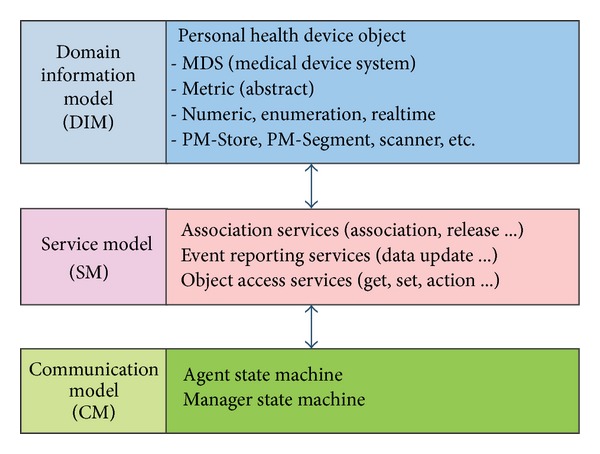
The X73 protocol stack of a sensor node.

The X73 standard defines the concept of Agent and Manager. The Agent represents the personal healthcare equipment (i.e., sensors), whereas the Manager represents the smart terminal equipment (i.e., the Sink in our system). The X73 healthcare middleware is mainly concerned with the communication interface and data exchange between the sensors and the Sink node. In the process of data transmission, an X73 software adapter is needed for the conversion of data formats, exchange protocols, and interface protocols. The X73 protocol stack of a sensor node is shown in Figure 5.

#### 4.1.1. Domain Information Model (DIM)

In the X73 standard, the Agent is expressed as a set of objects. Each object has one or more attributes. Attributes are used to describe medical data sent to the Manager by the Agent and to represent the Agent's various states, behaviors, and so forth. In addition to the attributes, the objects can also have a series of methods, such as GET and SET. The Manager communicates with Agents mainly through these methods. In addition, the Agent can produce a series of Events. When data in the Agent changes, it can invoke the corresponding Event to send the new data.

#### 4.1.2. Service Model (SM)

SM defines a flexible and efficient way for an Agent to send configuration information to the Manager. This way the Manager can create an Agent object. The Agent can report standard or nonstandard configuration information, which can be identified by the Manager during the state of association. In the process of configuration and information processing, the Manager will ask the Agent to describe all objects. Depending on the requirements of different applications, the Agent may vary from a very simple to a very complicated one. The Manager can save all the objects of an Agent through the Map object. This way it provides a plug-and-play functionality for the Agent. SM also defines the protocol used by an Agent to send measurement data to the Manager.

#### 4.1.3. Communication Model (CM)

CM defines the topological structure to enable one or more Agents to connect to a single Manager in a point-to-point (P2P) fashion. The IEEE 11073 standard is independent of the transport-layer protocol and assumes that an Agent and the Manager establish a connection by means of the Bluetooth or ZigBee standards. For each P2P connection, the internal system is defined by a Finite State Machine (FSM). The FSM defines the state and substate of the interaction between an Agent and the Manager, for example, the state of connection, association, and operation. CM also defines the entry, exit, and error information that may occur during the process of data transmission.

Taking into account that Java provides an efficient and hardware-independent platform for building both enterprise applications and portable-devices applications [25], it was selected for the implementation of the X73 protocol stack in order to provide system uniformity, distribution, and portability. The main modules include an ASN.1 codec library, Medical Device Encoding Rules (MDER), a message sequence mechanism, and FSM. In addition to the Adapter used for non-X73 sensors, a Gateway Agent sensor adapter was implemented by using the rules engine, which supplements the X73 uHealth system with a distributed processing ability and personalized features through embedding a lightweight Java-based Multiagent System (MAS).

### 4.2. uHealth Data Processing Subsystem Based on Cloud Architecture

**Figure 6:**
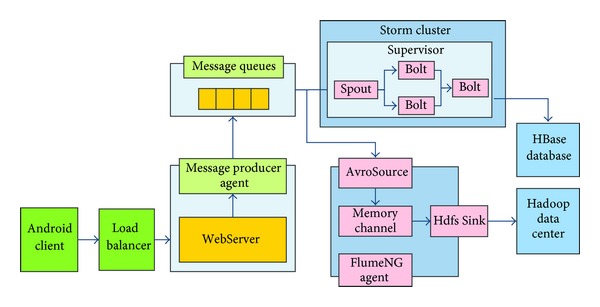
The distributed data processing subsystem of the X73 uHealth system.

In order to process the high volume of medical message flows in the cloud, an efficient storage strategy has been developed and implemented with a O (1) disk storage cost. The proposed cloud architecture consists of a distributed message module, a distributed real-time computing module, and a distributed log collection module, which are implemented based on Kafka [26], Storm [27], FlumeNG [28], and Hadoop [22].  Figure 6 shows the architecture of the distributed data processing subsystem in the X73 uHealth system.

#### 4.2.1. Message Queues Module

In the X73 uHealth system, it is necessary to develop a high-throughput distributed message queues module to satisfy the high volume of user's requests. Kafka [26] is an open-source software, which improves the processing performance and extensibility by lowering the complexity of the message queue system and thus meets the requirements of the X73 uHealth system. Kafka uses ZooKeeper [29] to coordinate and manage the relationship between the producer, broker, and consumer. Besides, Kafka implements an automatic load balancing technology based on the Zookeeper.

#### 4.2.2. Real-Time Computing Module

In the X73 uHealth system, an efficient computation of a complex medical data is often needed in a timely manner. For instance, the system should be able to process messages in the range of one million per second. Storm [27] can meet this demand. It is an open-source, distributed, and fault-tolerant real-time computing system. Storm receives real-time medical data from the message queue, compares it with various predefined health parameter thresholds, and monitors the patient's state of health in real time. When one of the medical parameter values continues to deviate abnormally, the system can intelligently send a health-warning information to the patient and concerned family members for the purposes of disease prevention and illness treatment in real time.

#### 4.2.3. Log Collection Module

In order to minimize the potentially large amount of log data in the X73 uHealth system, a robust and reliable fault-tolerant distributed log collection module is required. FlumeNG [28] is a reliable open-source, log collection system, which can satisfy this system requirement. FlumeNG serves as a link between the distributed data collecting component and Hadoop data center in this system.

#### 4.2.4. Hadoop Data Center

**Figure 7:**
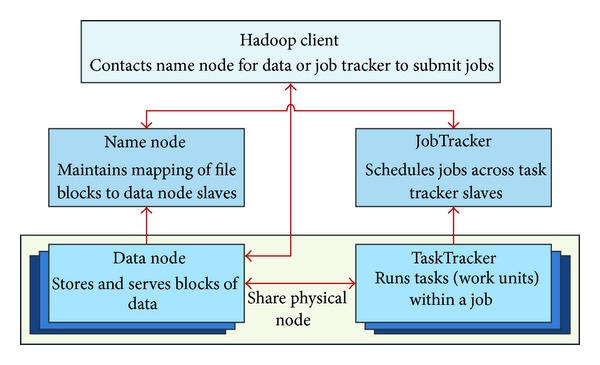
The X73 uHealth Hadoop cluster.

The X73 uHealth Hadoop cluster includes one master node and a number of slave nodes [30]. The master node consists of a Name Node and a JobTracker; the slave node consists of a Data Node and a TaskTracker.  Figure 7 shows the structure of the X73 uHealth Hadoop cluster.

## 5. System Implementation

### 5.1. X73 uHealth Sensor Node Implementation

As explained before, the X73 standard is mainly composed of three parts—DIM, SM, and CM. The following subsections provide implementation details of each of these.

#### 5.1.1. Domain Information Model (DIM)

**Figure 8:**
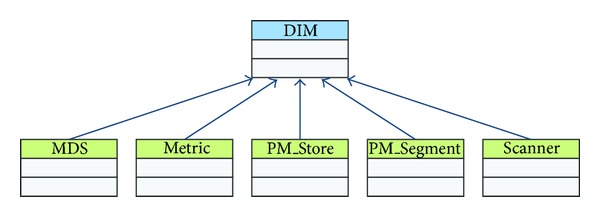
The X73 DIM class diagram.

DIM defines the object model of the X73 standard. In our implementation, DIM is an abstract class, which includes MDS, Metric, PM_Store, PM_Segment, and Scanner subclasses.  Figure 8 shows the UML diagram of DIM.

MDS represents specific medical equipment. For instance, its subclass MDS_10404 represents a Pulse-oximeter. One MDS uniquely identifies an Agent in the entire system. The Agent provides information to the Manager by the MDS internal attributes. MDS contains some Metrics, Persistent Metric Store (PM_Store), and other objects. The Metric class is the base class which is used to represent all the measured values, Agent status, and contextual data object. PM_Store is the data collected by the Agent, which is composed of metadata objects and PM-Segment objects. Persistent Metric Segment (PM_Segment) contains metadata and zero or more entity objects. Each entity object consists of one or more measurement elements. The Scanner observes the measurement data, encapsulates it to an event, and sends it to the Manager.

#### 5.1.2. Service Model (SM)

SM is used to exchange data between an Agent and the Manager. Message exchange and command execution mechanisms are provided, that is, the connecting and disconnecting messages, behavior message, data message, event, and so forth.

#### 5.1.3. Communication Model (CM)

**Figure 9:**
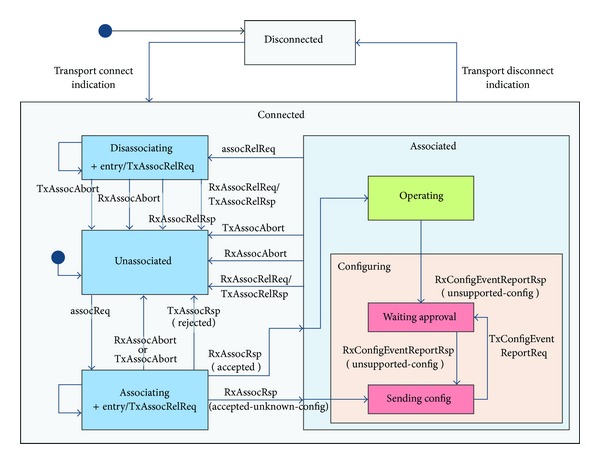
The state transition diagram of the X73 FSM [31].

CM defines the communication between an Agent and the Manager, which is an important part in the X73 system. A FSM is used to describe the state transitions of the Agent and Manager (Figure 9). Another role of CM is to change the DIM's abstract data model to transfer syntax. With the Medical Device Encoding Rules (MDER), mapping of DIM Java objects to an ASN.1 binary format (or vice-versa) is achieved.

The state class is the parent class of the Disconnected and Connected states. When an Agent is initiated, it is in Disconnected state. This state identifies that the connection between the Agent and the Manager has not been established yet. After connecting, the Agent will receive a Transport Connection Indication from the transport layer and the state changes to Connected. As long as there is an established transport connection, the Agent would be in Connected state all the time. This state includes four subclasses—Associating, Disassociating, Unassociated, and Associated. When the Agent wants to release the current association, it would send an Association Release Request to the Manager, and then will enter the Disassociating state. In the case of timeout, the Agent will send an Abort Request to the Manager, and then will enter Unassociated state. If operating normally, the Agent would always be in Associated state, which includes two subclasses—Operating and Configuring. If the Manager recognizes the configuration information sent by the Agent, it will send an Accept Response back to the Agent. Then the Agent enters the Operating state. When the Manager cannot identify the configuration information, it will send back an Accepted-Unknown-Config Response, and the Agent will go to the Configuring state.

**Figure 10:**
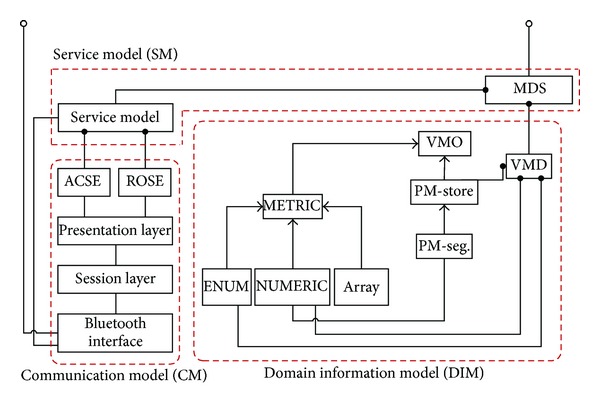
The relationship between DIM, SM, and CM in the middleware.

The relationship between DIM, SM, and CM is depicted in Figure 10.

#### 5.1.4. Rule Engine

**Figure 11:**
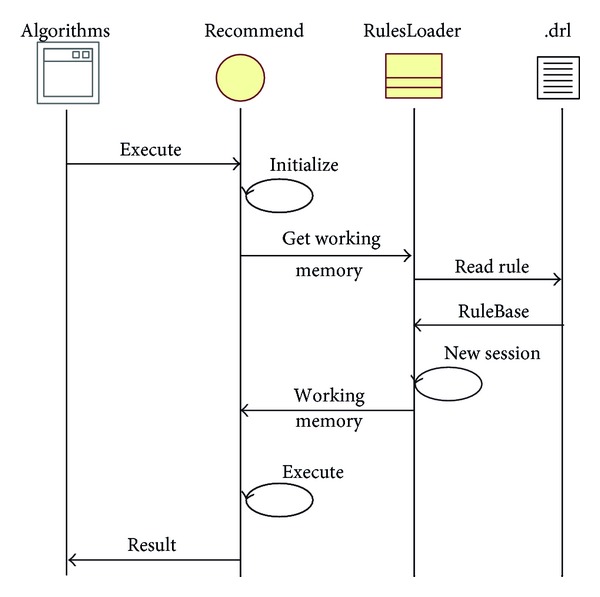
The Drools operation in the X73 Sink node.

Drools [32] is an open-source Java rule engine platform developed by JBOSS (http://www.jboss.org/overview/) and enhanced by implementing the Rete algorithm for object-oriented systems with the forward chaining excitation method. It is well suited to act as a rule engine API in this middleware.  Figure 11 shows the Drools operation in the X73 Sink node. With this interface design pattern, when a new rule is introduced in the system, the latter does not need to be recompiled. This ensures loose-coupling of the system.

#### 5.1.5. Gateway Agent

**Figure 12:**
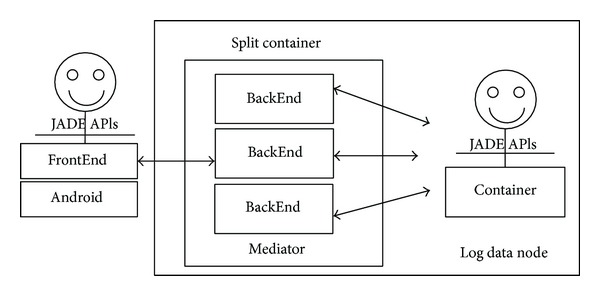
The JADE architecture of the X73 Sink node.

The Java Agent Development framework (JADE) [33]—a FIPA specification compatible open-source software framework—is adopted to implement the X73 uHealth system as a MAS with a distributed ability. An agent platform is running on the log data node (c.f. Figure 2). It provides an agent management system (AMS), a directory facilitator (DF), and message transport services. The gateway agent operates in mediator mode, that is, the JADE container is split into a front-end (running on the mobile personal server) and a back-end (running on the log data node).  Figure 12 shows the JADE architecture of the X73 sink node.

### 5.2. BASN Data Processing Subsystem Implementation Based on Cloud Architecture

The main goal of using a distributed message queue in this architecture is to free up the web server from the analytical data processing and objects creating, which are both time- and resource consuming. The traditional Message Queue system is mainly a mutual integration among enterprise applications, but the amount of data produced is not particularly big. In the system described here, the Message Queue system consumes the “big data” sent by (Android) clients in a timely fashion, in order to achieve very high performance and throughput. Kafka and FlumeNG are the main components of the system.

#### 5.2.1. Kafka

**Figure 13:**
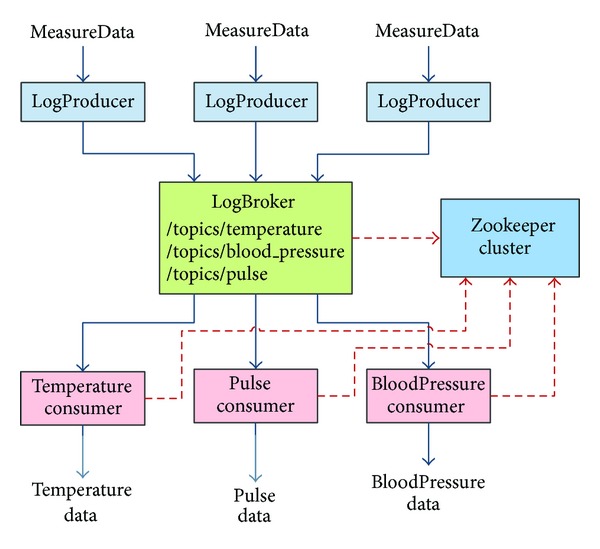
The use of Kafka in the X73 uHealth system.

Kafka performs a Buffer role between the web server and FlumeNG. The Message Producer is written in Java and deployed on a Tomcat server. The Producer processes data in an asynchronous nonblocking way. After receiving data from the Android client, the Producer would first cache it in the memory, and then when the triggering moment occurs or the number of messages reaches the preconfigured threshold, it would send all messages in a batch. The adoption of the asynchronous processing and the batch sending mechanism can greatly improve the Kafka system's throughput and network utilization.  Figure 13 illustrates the use of Kafka in the X73 uHealth system.

#### 5.2.2. FlumeNG

**Figure 14:**
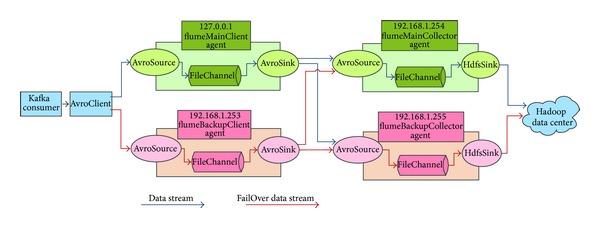
The architecture of FlumeNG in X73 uHealth system.

To guarantee reliability and fault tolerance of the whole system, the FlumeNG is built with a FailOver architecture. Every Kafka consumer runs a* flumeMainClient* Agent on a machine; at the same time another* flumeBackupClient* Agent runs on a backup machine. When* flumeMainClient* Agent fails, the client's data stream can be redirected to* flumeBackupClient* Agent transparently. The data collected from the* FlumeMainClient* or* flumeBackupClient* would be sent to the remote* flumeMainCollector* Agent. Similarly to the* flumeMainClient* Agent, the* flumeMainCollector* Agent also has a* flumeBackupCollector* Agent, which runs on a backup machine. In case of abnormal operation of the* flumeMainCollector* program, the* flumeMainClient*'s or* flumeBackupClient*'s data flow would also be automatically redirected to the* flumeBackupCollector* Agent. In addition, each Channel in the Agent is implemented with a FileChannel, so as to ensure that a message lost will not happen when the Agent operates abnormally.  Figure 14 shows the use of FlumeNG in the X73 uHealth system.

**Figure 15:**
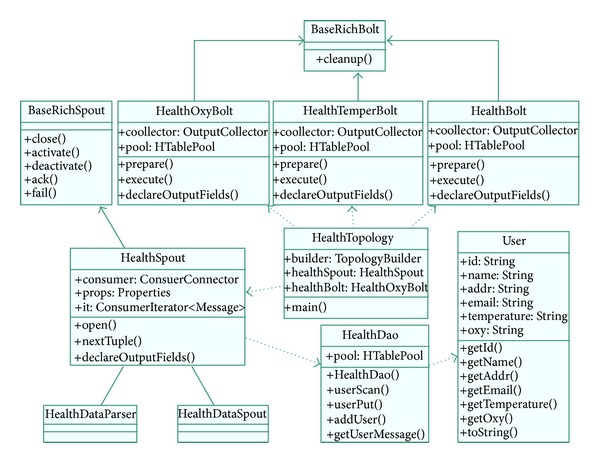
The UML Diagram of the X73 uHealth Cloud Architecture.

The UML diagram of the X73 uHealth cloud architecture is shown in Figure 15. The operation of the HBase is encapsulated into a* HealthDao* Java object, which provides APIs for the whole system thus making the HBase easy to use. In the Kafka part, the* healthSpout* is the key object. The consumer is initialed by this object with predefined properties. The output of Kafka is directed to Storm for real-time computing and to FlumeNG for saving. In the Storm part, a* HealthTopology* uses Map/Reduce to operate on the* Bolt* object and saves the results on the HBase database.

## 6. Results

### 6.1. X73 uHealth-Based Application

**Figure 16:**
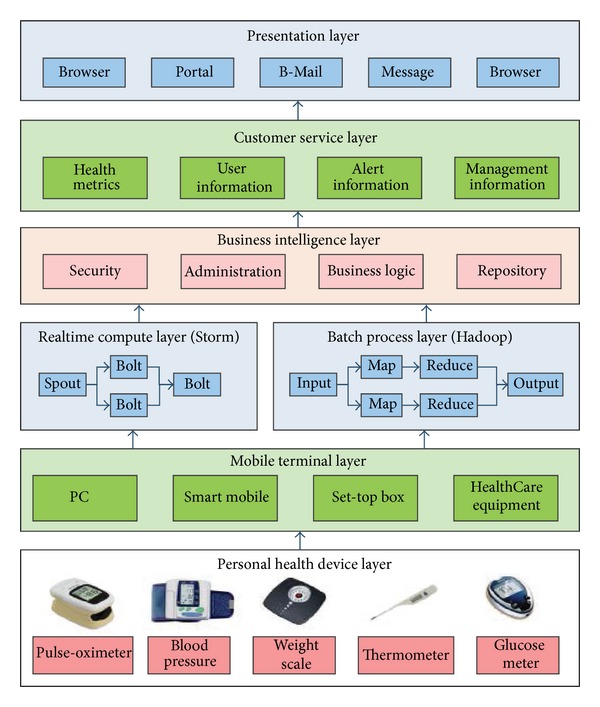
The architecture of the X73 uHealth application.

On the client side, various medical sensor nodes (such as pulse oximeter, blood pressure monitor, weighing scale, glucose meter, activity hub, etc.) send data to an intelligent terminal equipment, such as a mobile phone, a set-top box, and so forth, which provides an IEEE 11073 software adapter service. The gathered data is then transmitted to a remote cloud computing center. On the cloud side, the uHealth service provider may provide APIs for corresponding data mining, that is, searching for all kinds of potential medical threats or diseases and sending analysis information to doctors, community service staff, health and fitness service system, and so forth. The X73 uHealth based application consists of five layers (Figure 16).

**Figure 17:**
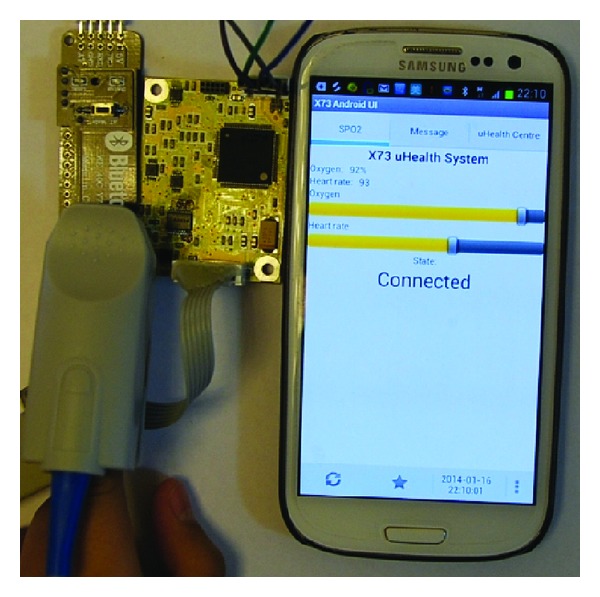
The X73 uHealth system testbed prototype.

The designed ISO/IEEE 11073 based healthcare middleware was tested on an Android mobile phone. A pulse oximeter was used as a medical sensor device (Figure 17). For communication with the middleware, an X73 adapter was used to ensure compatibility. In the future, a pure HTML5-compatible UI application will be developed to replace the Android application.

### 6.2. Performance Evaluation of X73 uHealth Cloud

To evaluate the performance of the X73 uHealth cloud component, a client generation of data was simulated and the results were collected through a script. The whole setup included two Kafka distributed message queue servers, two FlumeNG Agent servers, three Storm clusters composed of six servers, and a Hadoop cluster composed of six servers. Each server was installed on an Intel XEON PC with E5620 CPU and 8 GB memory. The message size was set to 300 bytes.

**Figure 18:**
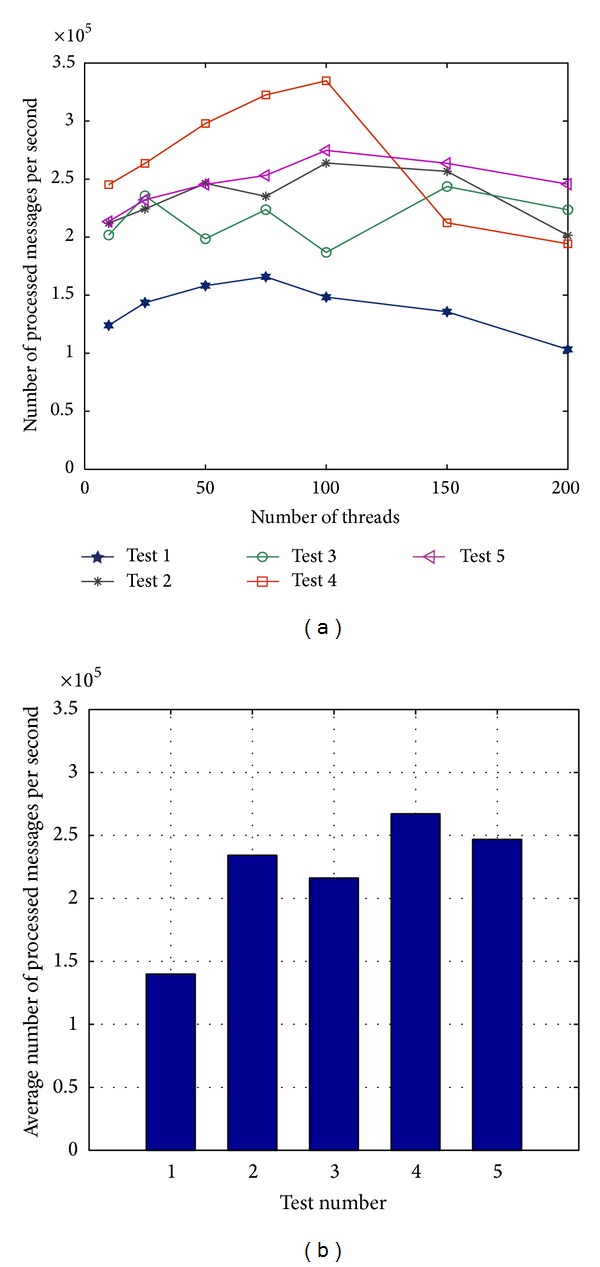
The performance of the X73 uHealth cloud: (a) the number of processed messages per second in each test for different threads; (b) the average number of processed messages per second for different tests.

During the tests, the clients send 10,000,000 messages to the cloud. The cloud thread concurrency (*X*) is 10 (1,000,000 messages/thread), 25 (400,000 messages/thread), 50 (200,000 messages/thread), 75 (133,333 messages/thread), 100 (100,000 messages/thread), 150 (66,666 messages/thread), and 200 (50,000 messages/thread), respectively. Test results are shown in Figure 18 for each of the five conducted tests (Table 1).

Test 1 shows that when the number of concurrent threads grows from 10 to 75, the throughput of the system is increasing. The maximum throughput reached is 165800 messages per second. However, for the number of threads more than 75, the system throughput gradually decreases to 103240 messages per second (with 200 threads).

In test 2, the number of Kafka Brokers and Storm Supervisors is two times that of test 1. The average system throughput reached is about twice the throughput of test 1, and the throughput slightly fluctuates with increasing the number of threads.

Compared with test 2, test 3 only uses one extra Storm Supervisor. The increase of the system throughput is not significant, but the fluctuation of the throughput becomes very obvious with increasing the number of threads.

In test 4, the number of Kafka Brokers is increased by one, compared to test 3. When the number of threads increases from 10 to 100, the system throughput shows an increasing trend. The throughput reaches a maximum of 334700 messages per second when the number of threads is 100. However, as the number of threads continues to increase, the throughput sharply decreases.

In test 5, the number of Kafka Brokers and Storm Supervisors is two times that of test 2. The system throughput, however, is not significantly increased compared with test 2, and the peak of throughput does not exceed that of test 4.

The above test results clearly demonstrate that the number of Kafka Brokers and Storm Supervisors used in the X73 cloud will influence the performance of the system. The optimal number of Kafka Brokers and Storm Supervisors will depend on the size of the clusters and messages used, and as such can be found for each particular case.

## 7. Conclusion

The design of a cloud-based X73 uHealth system has been presented in this paper. The system consists of a “big data” producer part and a “big data” consumer part.

In the producer part, a body sensor area network (BASN) produces a set of medical data for its corresponding patient. Each such network contains a number of small wireless medical sensors and a Sink node. To provide a plug-and-play environment for heterogeneous medical sensors which use different communication protocols, the system was developed based on the ISO/IEEE 11073 personal health data (PHD) standards. It works as a multiagent system (MAS) to provide intelligent collection of medical data from wireless sensors attached to the human body and subsequently to send the gathered data to a log data node based on the Always Best Connected and best Served (ABC&S) communication paradigm.

In the consumer part, a cloud-based architecture consumes the “big data” generated by the BASNs. It includes a Kafka-based distributed message module for caching of data by providing a load balancer and a high-throughput mechanism. A Storm-based distributed real-time computing module is used for data processing, providing real-time monitoring of the “big data,” and serializing useful data to an HBase distributed database. To save all medical data to the database, a FlumeNG-based distributed log collection module is used for storing the “big data” on a Hadoop data center.

The evaluation of the system performance confirms the system's high throughput and scalability allowing the processing of larger amount of data by increasing the number of cluster nodes. In addition, the system supports real-time data processing for urgent alerting and offline data processing for data mining.

This study may serve as a practical use case of utilizing an open-source distributed framework for solving patients' health problems, and as a model to solve the problem of “big data” collection. It could be used as a reference for “big data” processing in other fields as well.

## Figures and Tables

**Table 1:** The number of Kafka Brokers and Storm Supervisors used in each test.

Test Number	Kafka Brokers (KB)	Storm Supervisors (SS)
1	KB = 1	SS = 1
2	KB = 2	SS = 2
3	KB = 2	SS = 3
4	KB = 3	SS = 3
5	KB = 4	SS = 4
